# “*Si Mis Papas Estuvieran Aquí*”: Unaccompanied Youth Workers’ Emergent Frame of Reference and Health in the United States

**DOI:** 10.1177/00221465221122831

**Published:** 2022-09-09

**Authors:** Stephanie L. Canizales

**Affiliations:** 1 University of California at Merced, Merced, CA, USA

**Keywords:** emergent frame of reference, Latinx youth workers, precarious labor, subjective health, unaccompanied minors

## Abstract

Relying on in-depth interviews and ethnographic data in Los Angeles, California, this study examines the health experiences of unaccompanied, undocumented Latin American-origin immigrant youth as they come of age as low-wage workers. Findings demonstrate that unaccompanied, undocumented youth undergo cumulative physical and mental health disadvantages in the United States’s secondary labor market and during critical developmental life stages while lacking the parental monitoring and guidance to navigate them. Developing comparisons between their past and present living conditions and between themselves and other youth in Los Angeles—what I refer to as an *emergent frame of reference*—youth workers come to perceive family disruptions, and especially separation from their parents, as the most salient factor affecting their health. While some youth ultimately resign themselves to short-term attempts to assuage illness, injury, or distress through activities like substance abuse, others pursue community connections and support groups that can sustain them long term.

Health scholars have long examined Latinx immigrants’ health relative to their U.S.-born counterparts. What is known as the “Hispanic” or “immigrant” paradox has been linked to immigrant selectivity and immigrants’ culturally informed health behaviors (Boen and Hummer 2019) as well as immigrants’ embeddedness in close-knit families and protective ethnic enclaves that buffers from health risks through ties that offer the material and emotional benefits of co-ethnic solidarity (Osypuk et al. 2009; Zhou and Bankston 1998). More recently, scholars of social, economic, and legal stratification have argued that immigrants can experience cumulative disadvantages (Riosmena et al. 2015) through differential health risks (e.g., living and work conditions, stress), a lack of resources to manage risk (e.g., income, social networks), and diminished access to health-promoting services (e.g., public benefits, health care; Castañeda et al. 2015; Suliman et al. 2009; Torres and Young 2016). Within these lines of research, the notable erosion in Latinx immigrant advantage over the life course and across generations has been explained by some as a product of immigrants’ individual behaviors and their “negative acculturation,” but by others who critique “victim-blaming explanations” (Viruell-Fuentes 2007:1525) as produced by contextual and structural factors (Riosmena et al. 2015; Viruell-Fuentes et al. 2012), such as embeddedness in resource-impoverished and/or exploitative institutions (Dollar 2019) like the United States’s secondary labor market. This study considers the health experiences of unaccompanied, undocumented Latin American immigrant youth as a component of their adaptation to life in the United States.

Increasing attention has been given to the rise of unaccompanied Latin American youth migration to the United States since the humanitarian crisis of 2014, but much of immigrant health research has focused on adult immigrants. When studying children and youth, the presence of parents or other adult caregivers that guide youth during the transition from adolescence to young adulthood—a critical period in the life course that determines later morbidity and mortality (Montez and Hayward 2011)—is assumed. In the United States, where this research was conducted, the term “unaccompanied youth” refers to minors (age under 18) who migrate without a parent or guardian and are apprehended by Customs and Border Protection, including minors separated from family units (Human Rights Watch 2019). Latin American minors migrate to the United States alone pursuing employment, education, and adventure as well as to flee war, famine, natural disasters, and separation from family members (Hernández-León 1999; Ressler, Boothby, and Steinbock 1988). Those who arrive in the United States in their teen years (13–17 years of age)—members of the 1.25 generation—are more likely to be unaccompanied by a parent or caregiver (Hamilton and Bylander 2020) and are among the most likely to experience direct entry into the workplace rather than schools (Canizales 2021b). Thus, unaccompanied teens have a “comparatively more problematic adaptation” relative to their younger immigrant counterparts (Rumbaut 2004:1191). The persistence of Latin American youth migration and the rising rates of unaccompanied immigrant youth resettlement in the United States make urgent the need to understand immigrant youth’s health experiences as they come of age.

While others have investigated the health experiences of unaccompanied minors apprehended at the border and formally resettled with sponsors (see Berger Cardoso 2018), I focus here on Central American and Mexican young adults (18–31 years old) who migrated as unaccompanied youth (11–17 years old), who were not apprehended when they crossed the U.S.–Mexico border, and who remained unaccompanied as they came of age in the United States. I use “youth” and “young adults” interchangeably in this study because conversations and formal interviews included recollections of migration when participants were minors. Additionally, participants referred to themselves in Spanish as “*jóvenes*,” which translates into “teenagers.” These youth arrived in the United States with prior work experiences after having worked alone or alongside their parents or adult caretakers in their home country (Woodhead 1998) and thus anticipated entering full-time employment in low-wage labor occupations. Youth’s unaccompanied and undocumented statuses, financial obligations across borders, and the high cost of living in Los Angeles, California, relegated them to exploitative work in the secondary labor market (Canizales 2021b) even as they aspired to attend school (Canizales 2021a).

In attending to the institutional (i.e., work and family) and life stage (i.e., adolescence) contexts that unaccompanied, undocumented youth occupied, this study analyzes how unaccompanied, undocumented, and underage immigrant statuses intersect to create distinct but cumulative health risks. And because research has found that perceptions of disadvantage, stress, and risk shape behaviors and practices that mitigate or exacerbate cumulative disadvantage (McLeod 2012), I consider how unaccompanied youth’s perceptions of the sources of and solutions to adverse health experiences determined their health experiences as it shaped their responses to financial, physical, and psychological stressors both in and out of the workplace.

Findings show that unaccompanied immigrant youth workers faced an array of exploitative and unsafe workplace conditions, including financial, physical, and emotional health stressors in the workplace that adversely affected their health. Stressors were exacerbated by what I refer to as an “emergent frame of reference” that develops over time as youth (1) compared life in Los Angeles to that which they left behind in their origin country, where they were accompanied by parents, siblings, and other family members, and (2) observed that their everyday lives were different from other Latinx immigrant and nonimmigrant youth growing up with parents and as students. Youth workers’ perceptions that their unaccompanied status contributed most greatly to their relegation to work and therefore their health experiences in the face of indefinite family separation led some to feelings of resignation and the pursuit of shorter-term interventions like substance abuse to assuage illness, injury, or distress. Others, meanwhile, pursued strengthened interpersonal and community ties that supported their adjustment and well-being in the longer term. These findings increase our understanding of how contexts of incorporation shape health experiences and outcomes across the life course and offer important insight into an understudied area of unaccompanied and undocumented immigrant youth’s lives with implications for immigrant health research and policy. I discuss these implications in greater detail in the conclusion of this article.

## Background

### Undocumented Latinx Immigrant Health: Risks and Protective Factors

By focusing on the experiences of undocumented young adults who grew up as unaccompanied youth workers, this study advances existing research that tends to examine the health experiences of adults and parents as heads of households and workers or adolescent and young-adult students in parent-led households and schools. In this work, scholars argue that the undocumented immigrant experience is plagued with health risks that begin at migration (Vogt 2018) and continue through the adjustment to a new society and culture (Portes and Rumbaut 2001). This includes navigating housing, neighborhood, and labor market conditions (Hamilton and Chinchilla 2001; Hondagneu-Sotelo and Pastor 2021) and experiencing blocked access to social support such as health care (Doshi et al. 2020). Together, these factors create material and social disadvantages that contribute to physical and psychological stressors or conditions that impinge on a person’s ability to maintain their baseline health functioning (Pearlin and Bierman 2013), with long-lasting health effects for individuals and families across the life course. Material disadvantages early in life (e.g., educational disadvantage, impoverishment, or family dissolution) can lead to disadvantages later in life (e.g., employment and housing instability, social isolation, or illicit behaviors; Hoffmann 2016). So too can early life psychological stressors like fear, depression, and anxiety wear on the body as people age through processes including developmental delays and heightened psychological reactivity (Torres and Young 2016). When persistent, stressors become chronic (Wheaton 1990), and they can become toxic when faced without the support—familial or otherwise—needed to buffer material and health effects (Shonkoff and Garner 2012).

With some exceptions (see Canizales 2021a, 2021b; Diaz-Strong 2021), research examining undocumented youth’s stressors has tended to focus on the mental and emotional health tolls of transitioning in social roles from student to worker (Gonzales 2015), from child to parent (Enriquez 2020), or in legal statuses from undocumented to DACAmented (Patler and Pirtle 2018). Common among them is the relentless fear of family separation through deportation (Dreby 2015). Left out of both undocumented Latinx worker and undocumented youth coming-of-age and incorporation research, the health experiences of unaccompanied, undocumented Latinx youth workers are important to consider because of the significance of labor market incorporation and work conditions for immigrant health (Dollar 2019) and the growing number of children migrating from Latin America to the United States in search of work each year.

Unaccompanied, undocumented youth migrating to the United States find work in the secondary labor market to support themselves and their left-behind families and encounter its many exploitative features (Canizales 2021b). Employed within the precarious labor regimes (Sassen 1996; Torres et al. 2013) of manufacturing, restaurant and service work, construction, and the like, unaccompanied immigrant youth encounter wage suppression and theft; absence of workplace benefits; the likelihood of exposure to occupational hazards such as physical strain, dangerous heights, and performing repetitive motions; and sexual and gendered violence. Like undocumented adults, undocumented youth are compensated less for their work in hazardous settings relative to their documented counterparts and are often silenced in cases of grievances or rights violations (Gleeson 2016). Limitations to occupational mobility and workers’ rights imposed by race, class, gender, and legal status positions can also increase risk for injury or illness, creating lasting and irreversible impacts on immigrants’ physical and mental health. Undocumented workers are also subject to chronic stressors from interpersonal violence, stigma, and discrimination (Del Real 2019; Garcia 2018), which wear on the body through physical ailments and emotional distress. It is under these structural and contextual constraints that unaccompanied youth workers’ health experiences are produced and their health outcomes determined.

### Immigrant Youth Health, Caregivers, and Social Incorporation

Notwithstanding the health risks faced by immigrants, incorporation theorists agree that ties among families and extended kin and with “significant others” have meaningful effects on youth’s coming-of-age trajectories, including their health (Portes and Rumbaut 2001; Zhou and Bankston 1998). While the presence of adults and opportunities for institutional health access are no guarantee of better health outcomes, these studies suggest that they often have a buffering effect for immigrant youth, which durably benefits their well-being and social, economic, and mental health. The accessibility of support can determine whether an event produces resilience or trauma, with the presence of supportive networks promoting resilience (i.e., the ability to adapt to stressors) and their absence resulting in trauma that affects social integration and well-being. Indeed, studies of refugee (Luster et al. 2010) and foster (Greeson 2013; Perry 2006) youth find that a supportive adult figure and caregiver is a critical source of material and emotional support that promotes better health, self-concept, and overall well-being of children separated from biological parents.

While forces of globalization, racial capitalism, and neoliberal immigration policies lead to Latin American-origin youth’s displacement and migration, their unaccompanied and undocumented statuses in the United States obligate financial, social, and emotional independence (Canizales 2015; Martinez 2019). Prominent frameworks of immigrant youth incorporation (including health as an indicator of incorporation), like segmented assimilation theory (Portes and Rumbaut 2001), do not account for youth’s independence. Instead, researchers have focused on how parents and caregivers often provide financial support that allows children access to doctors (however limited), while schools can be health-promoting institutions, and youth’s institutional positions in parent-led households and school contexts offers several important sociocultural protective mechanisms.

Youth’s limited financial resources are offset by immigrant parents, typically mothers, who leverage their social ties and social capital to patchwork together community resources (Gomez Cervantes and Menjívar 2020). Even with parents as primary financial providers, youth who work part-time to supplement household income might experience stress related to role and time management and pressures associated with brokering (Delgado 2020). They may also benefit from a sense of autonomy, responsibility, and resilience (Estrada 2019). Outside of protective parent-led households and school contexts, unaccompanied, undocumented youth workers not only encounter distinct health risks but also lack the sources of support that youth are thought to have access to according to the literature. Furthermore, immigrant youth workers may face time and financial resource constraints in accessing formal health care services when they are available (Canizales 2021b) and rely on alternative remedies to illness, injury, and distress.

### Immigrants’ Perceptions of Health and Well-Being

Unaccompanied youth’s perceptions of their health experiences are important because individuals make decisions and develop practices to navigate adversity based on the meanings they derive from interpersonal and institutional interactions (Canizales 2021a; McLeod 2012). This includes undocumented populations, who are affected by policies that block access to health care but also experience stressors from perceptions of discrimination, stigma, and social, economic, and health threats. Together, these contribute to adverse health experiences and outcomes (Torche and Sirois 2018).

Perceptions of family, friend, and community support are also robustly associated with higher levels of self-reported physical and mental health for Latinos (Mulvaney-Day, Alegria, and Sribney 2007). These perceptions are shaped by the frames of reference that people rely on to make sense and meaning of their trials, triumphs, and futures in the host society (Waters 1999). First-generation immigrants assess their ongoing experiences in the United States in a binational frame of reference, comparing the life they left behind with the experiences of family members and compatriots still living there (Foner 2000). The 1.5 and second generation—immigrant children and children of immigrants—are thought to be less likely to have a binational frame of reference because of their limited or secondhand knowledge of their countries of origin (for exceptions, see Fernandez-Kelly 2008; Louie 2012), leading them to instead compare themselves with peers in the United States (Abrego 2014; Gonzales 2015), which can produce feelings of privilege or disadvantage.

Unaccompanied, undocumented youth workers who migrate during adolescence, like the study participants considered here, retain varying degrees of origin-country ties and memories (Canizales 2019; Diaz-Strong 2021). Having been workers in their home countries and lone child migrants to the United States, unaccompanied, undocumented youth workers remember well both the lives and relationships they left behind and the migration journey that forced family and community separation. Hence, the negative impact of the structural conditions of undocumented low-wage labor compounds with an emergent awareness of the absence of such stressors for immigrant youth with parents that heighten feelings of loneliness, produce emotional harm, and exacerbate financial and physical health disadvantages. The frame of reference from which youth workers make meaning of their living conditions, work experiences, and health emerges from firsthand or secondhand exposure to the experiences of immigrant youth, both with and without legal status, who grew up with caregivers and were primarily students. Study participants identified their unaccompanied status as having relegated them to low-wage work and being the main source of their adverse health experiences. Youth’s health behaviors and practices reflected their efforts to assuage loneliness, fears of navigating an unknown social *sistema* (system), and longing for intimate kinship ties.

## Data and Methods

The findings presented here came from a larger study consisting of ethnographic and interview data that were collected from the Pico-Union and Westlake/MacArthur Park neighborhoods of Los Angeles, which are two miles west of Downtown Los Angeles and primary sites of residence for Latinos. These data were gathered across four years with low-wage youth workers who participated in support groups, church youth groups, and community cultural events throughout Los Angeles. I gained access to these groups and events through previous research in which I investigated how participation in community organizations shapes unaccompanied, undocumented youth’s incorporation pathways (Canizales 2015, 2019). Important to the analysis here is my four-year long participant observation with an informal support group, *Voces de Esperanza*, herein *Voces*. My introduction to the group of unaccompanied youth workers was facilitated by its two coordinators, Wilfredo and Jorge. Together, the two guided youth through weekly open-ended conversations in response to one simple question: “How was your week?” Youth described the space as one in which they could find community and “*desahogar*” (unburden) from stressors faced in their work, school, family, and community lives. At *Voces* and other fieldsites, I made formal announcements and had informal conversations to recruit interview participants. Initial interviews began with members of groups I observed, but referrals by interview participants expanded the sample. Using a snowball sampling strategy, I completed a total of 75 in-depth, semistructured interviews with Central American and Mexican young adults, ages 18 to 31, who arrived in Los Angeles as minors (ages 11–17) between 2003 and 2013. Seventy of the 75 total respondents were full-time workers upon arrival in the United States, although 2 participants were unemployed at the time of our interview. Five of the participants were financially supported by long-settled relatives upon arrival and are therefore not categorized as full-time workers. Hence, I focused on the 70 participants who were full-time workers here.

Due to their urgent need for housing and employment (Hagan 1998; Mahler 1995), unaccompanied, undocumented youth tend to enter low-wage occupations within days of arrival in Los Angeles. Most study participants worked in three occupations: garment manufacturing (n = 30; 43%), restaurant service (n = 10; 14%), and domestic and janitorial work (n = 8; 11%). The rest (n = 20; 29%) worked in occupations such as construction, carwashes, mechanic shops, day laborers, and the like. Two were unemployed at the time of our interview but grew up as full-time workers in Los Angeles. Although unaccompanied youth workers do not enroll in the K–12 educational system, some do attend adult schools for English as a second language, and 22 (32%) of the full-time workers in the study sample went to classes for a few hours each day, Monday through Thursday.

Of the 70 full-time workers at the center of this study, a majority (84%) arrived in Los Angeles from the Central American countries of Guatemala (n = 48), El Salvador (n = 9), and Honduras (n = 2), while 16% were from Mexico (n = 11).^1^ Of the Guatemalan respondents, 36 (75%) originated from rural highland communities and identified as indigenous. These young people grew up speaking the indigenous languages of K’iche’, K’anjobal, Mam, or Akateco—just 4 of the 21 Mayan languages spoken in Guatemala—and learned Spanish as a second language in their home communities or in the United States. Reflecting the historical dominance of adolescent males in unaccompanied youth labor migration flows (United Nations High Commission for Refugees 2014), the youth worker interview sample included 51 men and 19 women. The median age of interviewees was 23. The median age at migration was 16, with the youngest participant migrating at 11 years old and the oldest at 17. Each interview participant received a cash incentive preapproved by the University of Southern California Institutional Review Board. When describing workplace conditions, I relied on participants and others in the field because I was unable to enter garment factories, private homes, restaurant kitchens, and the like due to the potential harm to study participants.

Data collection included taking notes during field observations and typing detailed field notes of observations and interviews immediately after leaving the field. I took cues from my surroundings while taking notes. For example, when attending a support group or book club meeting or in settings where others took handwritten notes, I jotted my observations into a small notebook; in other cases, like community cultural events where participants took photos or accessed social media on their phones, I used the notes feature in my iPhone to record observations. In all cases where notes were taken, important quotes were written in their original language (English or Spanish) to maintain their original meanings (Emerson, Fretz, and Shaw 2011). When the setting did not allow for notetaking, I audio recorded my observations after leaving the field, typically in my car. Each interview for this study was conducted in Spanish and audio recorded with the consent of the participant. Recordings were transcribed verbatim, and only the portions of the Spanish-language interviews reported in this article were translated into English.

Fieldnotes and transcripts were coded in Dedoose, a qualitative data analysis software. The theme of unaccompanied, undocumented youth’s health experiences emerged as I began coding using a flexible coding approach (Deterding and Waters 2018), which involves indexing the texts and applying analytical codes informed by recurring themes. These themes were based on the existing immigrant integration, labor, and health research as well as from the original data. As I coded for “wage theft,” “unstable work,” and “harassment at work,” which I later merged into the code “workplace violence,” for example, I began to note how these conditions operated as “financial,” “physical,” and “mental/emotional” stressors individually, which then interacted psychosomatically. I analyzed youth’s responses to my interview questions about whether their labor or living conditions would change if they attained legal status, linguistic proficiency, and/or had a parent present. To my surprise, many responded negatively to the question about legal status but positively to the issues of language and parental presence. Observational data in the support group, church gatherings, and other community spaces included youth frequently describing the value of interpersonal and institutional ties. This analysis contextualized the initial “coping mechanisms” code that was later disaggregated into “short-term” and “long-term” coping strategies to feelings of “emotional distress” (e.g., “loneliness,” “fear,” “anxiety,” “stress,” “depression,” and “overwhelming emotions”). I explore the relationship between these themes in the following with attention to how perceptions of the cause of stressors motivated health behaviors.

## Results

### Unaccompanied, Undocumented Latinx Youth Workers’ Stressors

Unaccompanied, undocumented youth workers occupy intersecting marginalized social locations, which combined to produce distinct health experiences and outcomes. For undocumented youth growing up unaccompanied, surviving requires navigating life in the United States independently to make ends meet through secondary labor market employment (for a detailed analysis of how gender and ethnoracial identities shape unaccompanied youth’s employment and mobility prospects and everyday work experiences, see Canizales 2021b). Not only are youth workers responsible for their own survival, but the financial strain of providing for left-behind families also weighs on them. Hence, being unaccompanied shapes health by placing responsibilities on youth’s shoulders that are typically thought of as reserved for adults in Western societies (Canizales and Diaz-Strong 2021), exposing them to physical and mental health risks and resources. While the intricacies of the health risks of work vary by industry, participants shared similar experiences of physical illness and injury; the mental and emotional distress of verbal abuse, name-calling, and financial stress; and difficulties escaping these conditions.

Youth across occupations reported not taking rest or meal breaks; feeling back, neck, and leg cramps throughout the day; lacking adequate safety training or materials; and being exposed to dangerous heat, chemicals, and sounds for up to 12 hours per day, leading to headaches, bloody noses, and eye, neck, and back aches and tensions (Canizales 2021b). Much of the pain they endured stemmed from the repetitive nature and extended hours of their work. These pains lingered in youth’s bodies and hindered their full participation at work and in other spaces such as language school, church, and community events (Canizales 2015, 2019). For Martina,^2^ a 27-year-old who migrated and began working in the garment industry at age 14, her work in manufacturing had several physical effects:

There are jobs that you have to do a lot of [physical] movement . . . I feel so tired. You also feel mentally exhausted. It’s 12 hours of the same sound in your mind. Then you get to class, and you still hear the same sound in your mind. You spend 12 hours [at work] then another 2 hours sitting [in class] aside from the 12 hours at work. If you count what time people start [work] at 6:00, 7:00, 8:00, 9:00 [a.m.], [and then you spend] another three hours at school. You spend 12, 13, 14,16 hours [just] sitting.

Like Martina, other undocumented youth workers often endured exhausting jobs and experienced wage theft but persisted in exploited positions because of transnational financial urgency (Canizales n.d.). Ultimately, these jobs led to new forms of physical and mental health stressors.

Carlos, a 21-year-old who migrated at age 15, explained that when he clocked into work at three or four in the morning, the timecard “*automáticamente tira las 7:30*” (automatically prints 7:30). Later in the day, when he clocks out at 5:30 p.m., “it automatically says 3:30 or 4:00. It doesn’t show you that I was working those hours. When inspectors come to the office, they see my name on the computer and that I worked 8 hours. They don’t see all the other hours.” He is compensated in cash for the hours missing on his timecard, but because they are not included in the hours accounted for by the timeclock, they are not compensated as overtime. As Carlos explained, “With that schedule, I am always tired. I always feel sleepy and my head hurts. I work many hours and they don’t pay me.” Wage theft is a common practice used to exploit undocumented workers to extract profits, yet the financial and physical weight falls on workers, and the toll is grueling. The work conditions that youth experience produce exhaustion, fatigue, and body aches, and these are exacerbated by the emotional distress of the undercompensation of youth workers’ time.

While the physical impact of performing demanding labor for long hours might not be immediately evident, some mental health impacts are. For example, I heard many participants in *Voces* discuss the compounding physical, mental, emotional, and financial costs of low-wage work. Among them was Omar, an 18-year-old who spent four years in Los Angeles as a garment worker. Omar worked about 60 hours a week, earning him just under $300. He often stressed that his work did not bring enough money to support him or his family back in Guatemala. Workdays consisted of him navigating assigned tasks under harsh working conditions as well as facing ridicule for his language and skill from non-Indigenous Central American and Mexican coworkers (Canizales and O’Connor 2022). In February 2014, Omar took to his Friday night support group to *desahogar* (unburden), discussing his week at work, school, and community. Surrounded by 13 other unaccompanied Guatemalan Maya youth workers seated atop creaky wooden chairs in a circle formation, Omar stood up, hung his head, and with tears streaming down his face began to explain that there were “thoughts that come to my mind that I can consciously control but there are others that I try to control, and they creep up on me.” He felt a deep sense of stress and dread that he was working as much as he was, but he was still not making enough money to make ends meet—and not enough to fulfill his promise to care for his mother and siblings in Guatemala. He continued by explaining that his inability to control these thoughts gave him a headache. With all of these thoughts coming, he said, he had to take breaks so that he did not “go crazy.”

As an undocumented garment worker, Omar was exploited through piece-rate wages, which meant that time spent not working equated to lost wages, which only added to his feelings of desperation. Another participant gently probed, “What is it that you think about?” Vividly describing intrusive thoughts and other symptoms reminiscent of a panic attack, Omar said, “It’s that . . . it’s just that I don’t know what’s happening inside of me.” He immediately began to cry. Before taking his seat again, Omar explained that he tries to think about it, he tries to figure it out, but he does not know what is “happening inside” of him. He worries about how far his thoughts could go.

While the support group coordinators, Wilfredo and Jorge, typically offered commentary or feedback to participating youth, it was not customary to ask follow-up questions in the support group setting. On this occasion, however, Jorge looked around and asked if someone had “*algo que ofrecer*” (something to offer). There was silence. Perhaps feeling guilty that he triggered an intense reaction from Omar, Aarón clarified that the reason he asked what Omar’s thoughts were was because he wanted Omar to identify the root of his problem, “especially if it is causing physical pain.” He said that “if it is causing you headaches then you have to be able to pinpoint where that pain is coming from so that you can treat it.” Silence befell the group until another participant, Benito, responded to Omar’s confession by explaining that he also felt aches throughout the day:

I get a huge headache Monday through Thursday. I felt bad because of this headache. It comes to me because of the sounds. It gets very hot, there is so much noise, and I am always thinking about what is going to happen tomorrow. “What am I going to do with this headache tomorrow?”

The everyday physical conditions of Omar’s and Benito’ labor, like that of youth workers who know that their work will not be appropriately compensated, destabilizes their mental health. In a feedback loop, the psychological distress returns to the body through headaches, rushes of blood, breathlessness, and other signs of anxiety. Omar’s fear of “going crazy” caused him to take more breaks, while Benito was pained and distracted by his worries about tomorrow. Born out of the structural violence of the secondary labor market, these ailments also affected youth’s participation in the workplace and their financial stability.

Some months later, in a conversation about financial responsibility, Enrique, a 22-year-old who migrated at age 16, shared with the group that he was regularly feeling stress about how little he earned and that he had started saving fastidiously a few months before our meeting. Enrique began feeling a sense of financial stability until, earlier that week, his mother had called from Guatemala to say that she had accumulated medical debt. Enrique was exasperated. As he spoke, he leaned forward in his chair, rested his elbows on his knees, and wrung his hands. He explained that he felt compelled to remit his meager savings to his mother to alleviate her debt, which left him with nothing. Much like Omar and Benito, Enrique described how this stress was causing psychosomatic symptoms of pain in his chest and left arm—demonstrating a feedback loop between mental and physical health distress. The pain made it difficult to breathe, he explained, deepening his distress about his health in the face of resource depletion. This could be, he worried, his own medical emergency, but he would now be ill-prepared to address it. He would need to start over to build savings for these kinds of personal emergencies.

Evidently, physical, psychological, and financial stressors operate individually but also interact through feedback loops that produce cumulative disadvantages. How do youth make sense of these health experiences? And how do these understandings shape their own health behaviors and practices?

### How Perception of Risk Shapes Youth’s Subjective Well-Being

In their home countries, youth often work alongside parents, with families sending children to school only if or when the household economy allows. Once in the United States, however, the social and legal distinctions between adults as workers and children or adolescents as students tend to disrupt this arrangement, and the absence of familial networks invokes emotional distress. This is exacerbated over time, as frames of reference emerge that cause youth to compare their everyday lives to those they left behind—including their past selves—and to immigrant or U.S.-born youth (students) with parents.

Participants explained that because they were workers in their home countries with either no or inconsistent schooling, where they were surrounded by similarly situated peers, “*uno no sabe*” (one doesn’t know) that education is compulsory for children and adolescents in the United States and that full-time formal employment is not common. As youth workers came of age, many observed that U.S.- and foreign-born youth typically live with their parents, attend school full-time, and eventually join the labor market only after completing their education. Youth learned this at work, where they were surrounded by adults; in churches, where youth met other teens who attended high school or were college-bound; or in public settings like parks, restaurants, or stores, where young people were typically accompanied by adults. According to Fernando, who migrated at age 15 and had been living in Los Angeles for seven years when we met, “*si mis papas estuvieran aquí*” (if my parents were here), he would have been kept out of the workplace entirely and likely moved into traditional high school instead “because as your mom and dad, well, they . . . maybe they would have said I should go to school and they would have [financially] supported me. Maybe then I would not have worked when I got here.” Fernando believed that the absence of a caregiver determined his workplace entry and that having his parents would have mitigated the suffering he endured as an undocumented low-wage worker. Because youth grow up as workers and come to anticipate migration as a rite of passage or natural progression of their work lives as they come of age, it was not working itself but living *and* working without parental support that was identified as the most unnatural condition of their lives.

Youth associated the absence of parental caregivers with material deficit but often spoke of their mother’s absence as a source of significant emotional distress while navigating settlement challenges, which reflects gendered social expectations that mothers are the caregivers of their families (Abrego 2014). The same bicultural frame of reference that emerged for Fernando caused Rolando to reflect on the lack of care he received in the United States relative to Guatemala. Although employed and earning higher wages, when he reflected on his mother’s care and its absence, his confidence about his decision to migrate waned. Rolando was 15 when he arrived in Los Angeles. At the age of 24, he explained,

When I got here in this country, yes, I felt like I needed my mom because, well, my mom cooks for us, she makes tortillas by hand and everything. Everything is homecooked and freshly made. But here, here it’s all microwaves. Since I don’t know how to cook, here it’s all microwaves.

Rolando longed for his mother’s care, evinced through the fulfillment of her patriarchal gender role, which he also left behind. In her absence, Rolando was responsible for his own care as an unaccompanied youth, which heightened his loneliness and added greater responsibility. He continued by saying,

And if I don’t go buy the food myself, well then, yeah, there isn’t anything. So, that’s when I start to think sometimes, “I was better off over there. Even if I ate much simpler foods, but we ate. We had *atole* [a hot, corn-based beverage], many different things. But here? No.” Over there, my mom made coffee, she made us *atole*, many things.”

Being a teenage boy in Los Angeles, Rolando associated care with being fed and nurtured by his mother through food and felt the absence of care in the United States relative to what he had in Guatemala.

Rolando’s emergent frame of reference moved him to reconceptualize what it meant to be well. Even though it was not lavish food that he craved, the simple foods were something he looked back on fondly and identified as an indicator that although he was not materially better off, he was perhaps emotionally better off in his hometown with his family. Rolando described life in the United States as being more individualistic, which made him sad and created a less caring social environment:

Here, it’s different. Here it’s everyone for themselves (*aquí es cada quien*). I think that’s why I got sad. Well, and since no one over there can give me advice, and no one here can give me discipline. It’s just me, by myself. I’m alone (*solito*) in my apartment. Sometimes just by myself. And sometimes when you close yourself up inside, you start to get a lot of things in your head. Like, one day I even thought to get together with the people hanging out outside. I thought there was no solution to my life here. There were no jobs. There wasn’t anyone to give me a job. I thought, “Well, I probably won’t work and then I cannot pay my debt.” I said, “I am going to be on the street with these people.” But I thank God that I am not on the street.

Rolando began applying for jobs and through networks at his existing job was able to acquire additional manufacturing work. Rolando’s telling—in which he jumps from lacking advice to being lonely and work conditions—demonstrates the interconnections between conditions at work and emotional distress. It also evidences the growing feelings of hopelessness and resignation that come from find oneself “*solito*,” without advice or discipline, whether in the context of finding an apartment or taking on responsibilities like finding employment, paying off migration debt, and making ends meet. Rolando’s employment and binational comparative wage advantages seemed, in his case, to be emotionally outweighed by a need for care.

Dealing with the unevenness of family care between sending and receiving countries is compounded by uneven constructions of childhood across the two societies. Tomás, whose sister turned him away upon arrival in Los Angeles at the age of 14, described how comparing his life to that of other youth in the United States affected his mental and emotional health:

You feel discriminated against, like, I feel like less than them. I would look at other kids and say “Wow, why not me?” I would ask myself, “Why am I not a kid who was born here? Why aren’t my parents here? Why is my life different?” I say, “Look, the ones who were born here go to school, they have their parents, they have everything. . . . ” I wish I could speak the language, too. I feel like there’s no way out. I like being here, but I feel stuck. I feel less than others.

Tomás’s emergent frame of reference included comparing his experiences to those that he associated with youth born in the United States and the capital afforded by their parental ties. This meant they did not have to work like him and bred a sense of deprivation that caused him to be consumed with hopelessness, low self-esteem, and feelings of being stuck. At the age of 19, Tomás said that youth who migrate to the United States without their parents or familial support and who therefore grow up as workers “*vienen a sufrir*” (come to suffer). Just as discrimination based on race, immigration, and legal status determines health (Torche and Sirois 2018), Tomás’s case demonstrates that experiences of “discrimination” by family structure can also affect health outcomes (Russell, Coleman, and Ganong 2018). While having social support benefits mental and physical health, which can give a sense of meaning in life, youth’s feelings that they lack social support from a parent or caregiver can negatively affect their sense of self and their physical and mental health in turn. Developing binational and bicultural frames of reference as unaccompanied, undocumented youth workers in the United Steates requires youth to navigate their mental and emotional health, which is perhaps an unexpected aspect of labor migration and immigrant incorporation. As one participant put it in March 2014, “We, as children, we never feel what it’s like to be kids because we work to support our moms.”

Thus, while many unaccompanied, undocumented youth workers say that “*uno no sabe*” (one doesn’t know) that life as a child laborer is not a common U.S. experience, being in the United States exposes them to binational and bicultural differences in the roles of children, meanings of childhood, and transitions into adulthood that they might not have anticipated. In an April 2014 support group meeting, a young man explained a sense of disillusionment with the challenges that life in Los Angeles presented. He told the group that he was learning that after nine years in Los Angeles, “There are realities and there are fantasies. You feel pain from having left [your home country and family]. You feel pain *de no realizarse* [of not reaching your goals]. Everything, that made up that fantasy of what Los Angeles was when we are in Guate[mala], is false.” The atmosphere turned somber, as others hung their heads or stared across the room, appearing to reflect on the resonance of this statement. Breaking the silence and affirming youth’s disillusionment, the group coordinator, Wilfredo, responded, “*aquí no se está viviendo fantasia*” (we are not living a fantasy here).

### How Perceptions of Risk Shape Youth’s Health Behaviors and Practices

Time in the United States exposed youth to differences between their life with family in their origin country and their life as unaccompanied in Los Angeles as well as the differences in their lived experiences as low-wage workers and those of youth growing up with parents and as students. This emergent frame of reference informed their health behaviors and practices as youth began to seek out alternative remedies for illness, injury, and distress they faced relative to youth growing up with parents in the United States. Such remedies ranged from drug and alcohol use to the adoption of herbal supplements, spiritual healing practices, and exercise. It is important to note that the adoption of risky behaviors like drug and alcohol use are not coping strategies distinct to Latin American-origin and/or immigrant youth (see Patrick and O’Malley 2015). As youth workers grew up in Los Angeles, their social ties consist most immediately of their neighbors and coworkers—typically adult men and women who introduce them to their own coping mechanisms. The gendered nature of emotional expression—and the expectation that men do not have or share emotion, especially pain and sadness (Montes 2013)—shapes their access to health resources and adoption of coping mechanisms. Young women, for example, spoke of attending Zumba or dance classes or being invited to religious gatherings by coworkers and community acquaintances, while young men were more likely to be introduced to alcohol or other substances.

Fernando, introduced earlier as being disillusioned with the reality of lacking mobility prospects, became a weekly participant in the support group in 2013—one year after I began my observations. For several weeks after joining, he talked about his life before seeking support, when he spent nine years in Los Angeles “*perdido*” (lost) in a loneliness- and depression-induced state of alcoholism and heavy drug use. Fernando explained to the group in 2013, and to me later during an interview in greater detail, that he felt a deep pain because of family separation and especially separation from his mother. He struggled with tedious, unfairly paid work in the garment industry and had hardly enough money to pay the rent; his phone bill, which was especially important to be able to call his family in Guatemala on a biweekly basis; or to remit a sum of money that he felt justified the debt he accrued and the separation from his family in the first place.

The devastation of the pain of separation, the reality of exploitation and poverty, and disappointment in being unable to fulfill promises made to left-behind families compounded to compel some youth to rely on deadly practices to assuage or end the suffering they perhaps deemed inescapable. These forms of distress mounted to the point that three young men in support-group participants’ networks died by suicide in just a two-month period. I attended two of the vigils personally, and as family and community told stories to honor their lives, I heard time and time again that life in the United States was not what young people had envisioned for themselves. This disillusionment was “overwhelming” for many and led to the loss of life for some. Among the losses was a young man who hanged himself with a scarf from his doorframe in a Pico-Union apartment. His apartment faced a community garden where other youth spent their Sunday evenings “*cultivando*” (cultivating) plots of land to soothe their anxieties and reconnect with the land, as they had in their rural hometowns.

Often, the health resources that youth needed to *aguantar* (endure) and reduce the risk of unhealthy behaviors and poor physical health are at arm’s length (Umberson and Montez 2010), but the normalization and silencing of suffering across social ties can keep these resources hidden. When youth openly spoke about their shared challenges, they described being introduced to community-based health efforts or establishing groups along with their peers. For example, Justino, a 30-year-old who migrated at age 15, and Francisco, a 22-year-old who migrated at age 15, met at a garment factory where both struggled to keep up with piece-rate work. Justino learned of a karate class just outside Pico-Union that he began attending, enticed by a chance to be active and increase his stamina at work. He later invited Francisco. Although Francisco also enjoyed the physical activity and hoped to work at the karate studio, an eventual knee injury brought an end to his karate career. Wanting to maintain the “family” aspect of participating in karate, Francisco invited Justino to brainstorm what eventually became a recreational group. This group would meet on weekends and explore Los Angeles through hikes in the canyons and runs along beaches. Francisco shared, “We are a youth group meant to support other youth and teach them that there are healthy ways to have fun and invest our time. These sorts of things will serve us in the future because exercise not only helps you to be healthy, but it helps you with other things,” like the pace of work, productivity, and peer motivation. Justino and Francisco began the group as five “*compañeros*” (coworkers) who were also unaccompanied, undocumented youth. When I met the pair, their weekend crew included nearly 30 young immigrant women and men and was dedicated to planning outings and activities together.

Other youth countered the disadvantages of being unaccompanied by seeking emotional support, informally gathering with peers to “*desahogar*” (unburden) and let go of the “*timidez*” (timidity) and “*miedo*” (fear) that impinged on their ability to “*controlar los pensamientos*” (control their thoughts) and cultivate social ties. One way youth did this was through participation in weekly gatherings such as *Voces.* The aforementioned exchange between Omar and Benito in the February 2014 support group, for example, exemplifies how storytelling among youth can breed solidarity while also offering opportunities to share information. Just weeks after his tear-filled description of feeling like he was “going crazy,” Omar asked me if I practiced meditation. He later shared with the group that earlier that week he sat beneath a tree in a park near his jobsite during one of his self-prescribed breaks and mimicked the meditative posture of an older man nearby. Breathing deeply, Omar noticed his racing thoughts slow down, and he was empowered by what felt like a newfound control over his “overwhelming” thoughts and emotions. In sharing this meditation practice with the support group, he encouraged others to also take time to “*calmar los nervios*” (calm their nerves). Omar’s individual strategy informed the group’s collective health practice.

An emergent frame of reference made clear the importance of establishing guiding figures for youth growing up without parents. Youth learned strategies that they could pass on to others, thereby making them peer mentors, yet they also craved the guidance of adult figures. *Voces* coordinator, Wilfredo, along with other mentors from other community organizations had the ability to influence how youth organize—or think about organizing—their lives. Ernesto explained the influence that Wilfredo had on him:

When I came here? I didn’t come with a dream. I didn’t know anything about setting goals. I didn’t think about what I was going to do or where I was going to go. Well, I think, because over there [in Guatemala] they tell you that there is a little bit of everything here [in the United States]. I didn’t come with a dream and that affected me a lot because even today, this day, I have realized that I have wasted a lot of time because I didn’t have a direction. I don’t have anything. I have only worked.

Ernesto met Wilfredo three years prior to our interview, having been introduced by a friend after sharing that a broken relationship might have induced his depression. Ernesto recalled:

[My friend] took me to talk to him. Then [Wilfredo] told me we would keep talking. Sometimes I would forget to meet him, but he would call me, and I noticed that he encouraged me a lot. I started going to [*Voces*] and it is really encouraging to see people like yourself who are struggling, fighting, and studying.

Wilfredo and *Voces* represent one avenue for youth to become connected to mentoring figures and community organizations that counter their feelings of loneliness and isolation along with the lack of discipline and care that they experience.

Community mentors are individuals with whom unaccompanied, undocumented youth can share their ideas, thoughts, and confusions while also gaining tangible resources, social capital, and “*animo*” (encouragement) as they navigate U.S. economic, culture, education, social, and legal systems. Their mentors also provide tangible ties to organizations along with practices and beliefs that offer greater stability to the young person’s life. By attending *Voces* and engaging with Wilfredo, youth learned to leverage ideologies of individualism and self-responsibility to create intentional self and community care (Canizales 2015). This is especially important for youth whose emergent frames of reference incite feelings of being denied a normative childhood.

However, despite established coping skills, there are several limitations to the benefits and reach of these kinds of youth-led health groups and practices. For example, youth-led groups are self-funded and can create greater financial strain, while ethnoracial and gender hierarchies engender inequalities in accessing the benefits of organizational participation (for more on this, see Canizales 2015, 2019). Furthermore, individualized solutions do little to amend structural ills. Still, these examples are a testament to the complexity of youth workers’ social worlds. They demonstrate that youth can express agency, creativity, and flexibility in their efforts to attain health resources and that they can (re)shape their social environments, primarily through leveraging the interpersonal and institutional networks they acquire as they come of age. These examples also demonstrate how youth’s health behaviors and practices are informed by their perceptions that their primary health disadvantage is their unaccompanied status.

## Discussion and Conclusions

This study examined how unaccompanied, undocumented youth workers’ own understandings of their social positions affects their physical, mental, and financial distress and well-being and offered evidence of how this impacts their health behaviors and practices. Financially responsible for themselves in the United States and committed to supporting their left-behind families, unaccompanied youth enter precarious labor regimes made up of secondary labor market occupations riddled with hostile, unsanitary, and unsafe conditions. Importantly, though, while this work and their conditions have physical and mental health effects on youth, it is not necessarily the work alone that causes youth’s distress; rather, work compounds with youth’s *emergent frame of reference*—exposure to differences between life as workers with and without their families nearby and exposure to other youth who live what seem like entirely differently lives—that then breeds a sense of deprivation. A longing for parent(s) or caregiver(s) who can buffer the financial stress and physical and mental health harms exacerbates the distress caused by what is already exhaustive and exploitative work, demonstrating how precarity can be cumulative for unaccompanied, undocumented Latinx youth workers. Hence, youth identify family structure disruption—separation from their parents through migration and the absence of parents as expected primary caregivers in the receiving society—as a key cause of material and health disadvantages in the United States.

Youth’s emergent frame of reference related to their health informed their health behaviors and practices. Youth may find themselves overcome with a sense of hopelessness from the indefinite nature of family separation, especially in the face of restrictionist immigration policies and exploitative labor practices that make returning to their home countries (and families) out of reach. In these cases, short-term remedies such as the numbing effect of alcohol and drugs, or more drastic measures like taking one’s own life, become possibilities. But youth may also pursue short- and long-term remedies to balance their feelings of loneliness and hopelessness, cultivating community ties, engaging in physical and mental health group activities, and adopting collective health strategies. Others resort to short-term remedies like alcohol but over time come to adopt long-term remedies like starting or joining a youth group.

This research corroborates existing scholarship that argues that the immigrant advantage erodes over time and does so because of cumulative structural disadvantages (Riosmena et al. 2015). In the case of unaccompanied youth workers, possible health advantages decline as time in the secondary labor market ensures ongoing exposure to exploitation and workplace violence. In the United States, where childhood is considered “priceless” (Zelizer 1985), acknowledging children’s and adolescents’ presence in low-wage occupations should evoke policymakers’ concern and motivate collective action for workers’ rights. Organized struggle, particularly in the form of worker-led movements, might produce a sense of social membership while empowering workers, including youth, to challenge the precarious labor regimes that harm them (Torres et al. 2013), which addresses structural and subjective health risks to unaccompanied minors’ low-wage labor employment.

Policymakers and advocates might also act on behalf of unaccompanied youth workers by ensuring that they might pursue an education given that the emergent awareness that youth with parents/caregivers are more likely to be students produces feelings of deprivation and heightens emotional distress. Although youth workers cannot forego work entirely to attend school, state- and local-level governments might invest in English-language schools typically thought of as adult night schools to promote youth’s learning, especially because language learning has notable effects on youth’s employment and wage mobility (Canizales 2021a; Canizales and O’Connor 2021). Finally, policymakers and advocates should ensure mental and emotional health is prioritized in immigrant youth resettlement. These recommendations reflect the intellectual aim of this research to move explanations of immigrant health outcomes away from individual health behaviors and toward an analysis of the immigrant context of incorporation. New areas of health research open when we widen our view of undocumented young adults and unaccompanied minors as growing up in parent- or adult caregiver-led households and K–12 schools and consider the effects of parental absence and entry into labor institutional contexts during critical developmental years of the life course.

Important to consider here is that the physical and mental health experiences accounted for are indeed severe, but the lasting health consequences of trauma, injury, and stress suffered earlier in life may not be apparent until much later. Research shows that the health disadvantages of stress spill over into the lives of immigrant families and communities regardless of immigrant generation or legal status, and these can have serious consequences for subsequent generations (Del Real 2019; Enriquez 2020). Hence, a question these data leave unanswered is what the long-term physical and mental health consequences will be for unaccompanied, undocumented youth workers as they age and the generations that follow. Participants described injurious working conditions that are likely to persist, given their social/structural positions and the transnational nature of their financial needs and obligations. They also described immediate mental health challenges that crippled their social, financial, physical, and emotional health stability. While immediate health services are necessary for this population, researchers should also continue to explore long-term consequences beyond currently experienced headaches, eye tension, anxiety, depression, and panic attacks. This kind of chronic, toxic stress—alongside barriers in accessing and navigating care—is likely to lead to hypertension, diabetes, and other cardiovascular diseases as well as depression and anxiety. Participants have the advantage of time as young adults, but will the advantages be sufficient to counteract the harms identified in this study over the life course?

## Notes

1. The overrepresentation of Guatemalans is the result of my entering the field with a focus on Guatemalan unaccompanied minors. See Canizales (2015) for more on this.

2. All names are pseudonyms to protect participants’ identities.
